# Mechanical and metabolic interplay in the brain metastatic microenvironment

**DOI:** 10.3389/fonc.2022.932285

**Published:** 2022-08-18

**Authors:** Killian Onwudiwe, Alice A. Burchett, Meenal Datta

**Affiliations:** Department of Aerospace and Mechanical Engineering, University of Notre Dame, Notre Dame, IN, United States

**Keywords:** tumor metabolism, glycolysis, fatty acid synthesis, tumor mechanics, extracellular matrix, mechanotransduction, cell stiffness, brain metastatic microenvironment

## Abstract

In this Perspective, we provide our insights and opinions about the contribution—and potential co-regulation—of mechanics and metabolism in incurable breast cancer brain metastasis. Altered metabolic activity can affect cancer metastasis as high glucose supply and demand in the brain microenvironment favors aerobic glycolysis. Similarly, the altered mechanical properties of disseminating cancer cells facilitate migration to and metastatic seeding of the brain, where local metabolites support their progression. Cancer cells in the brain and the brain tumor microenvironment often possess opposing mechanical and metabolic properties compared to extracranial cancer cells and their microenvironment, which inhibit the ease of extravasation and metastasis of these cells outside the central nervous system. We posit that the brain provides a metabolic microenvironment that mechanically reinforces the cellular structure of cancer cells and supports their metastatic growth while restricting their spread from the brain to external organs.

## Introduction

Cancer genetic and metabolic aberrations are linked *via* oncogenes and tumor suppressors that play a key role in cell metabolism (1–3). They largely affect three major metabolic pathways: aerobic glycolysis, glutaminolysis, and one-carbon metabolism (4–8). These alterations make it possible for cancer cells to transition from simple adenosine triphosphate (ATP) production to the generation of large quantities of nucleotides, fatty acids, amino acids, and other intermediates necessary for rapid mitosis, proliferation, and cell growth (9–11). Cancer cells can even alter their metabolic programs to maintain cell-autonomous proliferation in the often nutrient-poor conditions of the tumor microenvironment (12).

Considering that cellular energy metabolism greatly impacts neoplasia and can determine cell fate (e.g., proliferation versus apoptosis), high energy metabolism in the brain could be largely responsible for the propensity and aggressiveness of metastasis to and within that organ (13). The brain is a highly vascularized structure, and its cells are largely dependent on circulating glucose for energy production (14). Normal brain cells derive most of their energy from aerobic oxidation of glucose, while metastatic cancer cells possess metabolic flexibility and depend not only on glucose for energy but also on glutamine and acetate, irrespective of their origin or subtype (8). This metabolic adaptation promotes the rapid growth of cancer in the brain (8).

Besides metabolism, the brain is also mechanically distinct from other organs. The increased energy demand in the brain tumor microenvironment supports hyper-vascularization and a leaky blood–brain barrier, which causes increased fluid pressure and increased shear stress within the tumor microenvironment (15, 16). The mechanical properties of brain tissue, such as its stiffness (i.e., Young’s modulus), are lower compared to other tissues in the body. This is in part due to the increased fluid pressure from leaky vasculature and excessive loss of cell mass in the brain caused by the tumor and chronic inflammation (16–19).

In brain tumors, there is a relatively elevated production of some ECM proteins (proteoglycans, hyaluronic acid, glycosaminoglycans, and collagen), which increase tumor stiffness and play an important role in tumor progression (20, 21). This is in part due to the increased metabolic stress and elevated YAP-TAZ signaling (20, 21). But unlike extracranial cancers that are typically stiffer than their host tissue, primary brain tumors (e.g., glioblastoma) are often more compliant than their surrounding tissues, as shown in **Table 1** (17). Mechanical forces are also strikingly altered in the presence of a brain tumor (28). For example, edema, which is a commonly observed clinical pathology, is a result of excess fluid pressure in and around brain tumors (29). Solid stress—exerted by growth-induced forces—from brain tumors is exerted not only within the tumor mass itself but externally as well, compressing the surrounding normal tissue, thereby reducing perfusion and inducing neuronal loss (30–32). These mechanical stresses are believed to be a major cause of the clinical symptoms seen in brain cancer patients (31).

**Table 1:** Stiffness of biological tissues in their normal and diseased states.

Tissue	Tissue stiffness (kPa)	Tumor Stiffness (kPa)	Reference
**Breast**	27	270	(22)
**Brain**	5.89	3.75	(23)
**Lung**	5	30	(24)
**Bladder**	3	8	(25)
**Liver**	6	12	(26)
**Pancreatic**	3	6	(27)

Cancer cells themselves have also altered physical properties; their mechanical integrity tends to be lower than that of normal cells in the brain and decreases with increasing tumor progression and metastasis (33–37). Tumorigenesis, for example, induces actin cortex remodeling, which in turn makes cancerous cells softer—a key advantage for uncontrolled division, migration, and infiltration. Reduced cell membrane stiffness enhances the ability of cancer cells to migrate from the primary tumor to secondary sites, with each organ hosting its own unique mechanical properties and forces (38).

**Figure 1:**
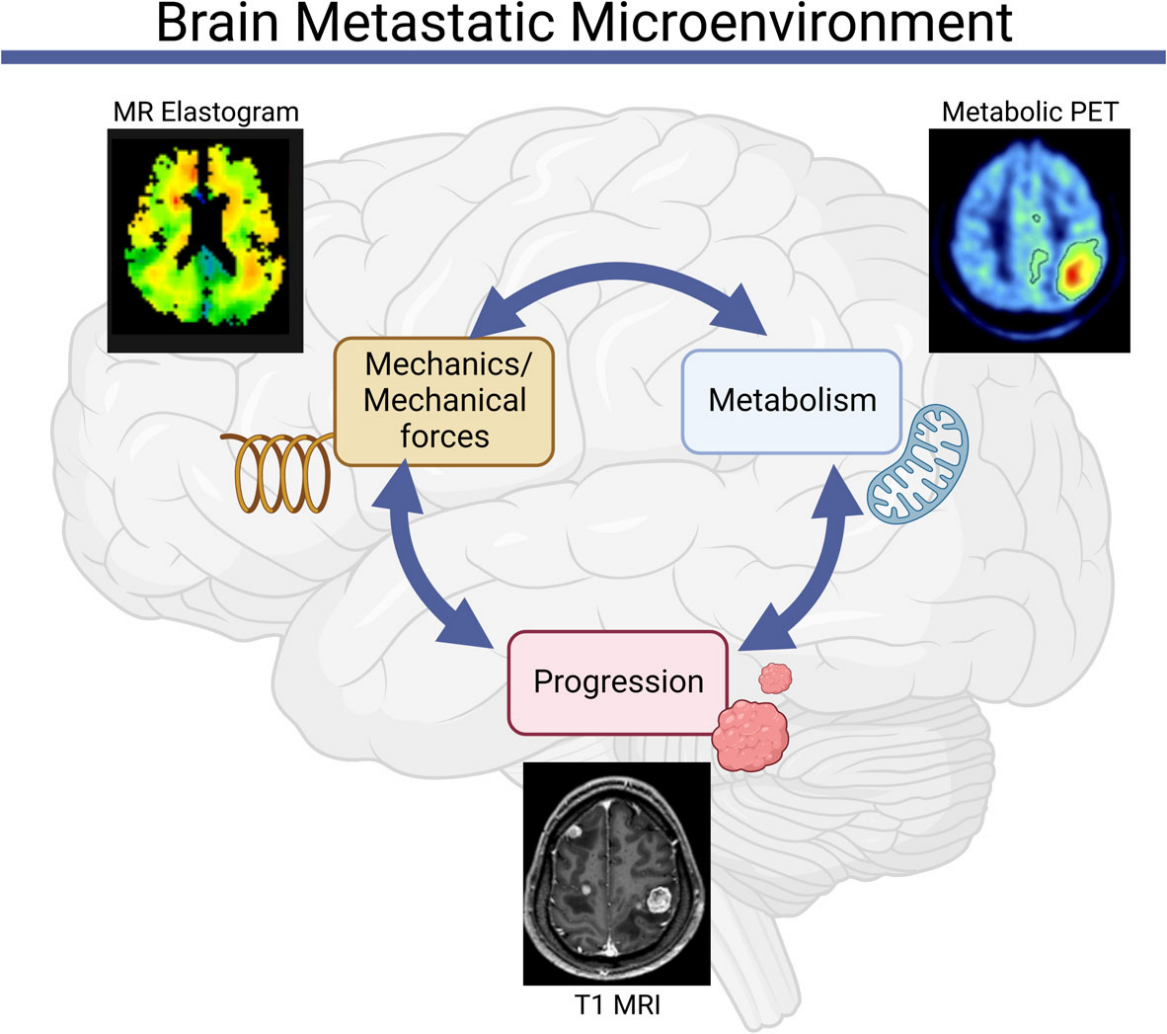
Mechanics and metabolism are linked in the brain metastatic microenvironment. Abnormalities in tissue and cell-scale physical properties influence and are influenced by metabolic processes, both of which can contribute to the initiation and progression of brain metastases [Clinical images reproduced from (39–41)].

In this Perspective, we explore how the brain microenvironment regulates unique relationships between metabolism and mechanics (**Figure 1**), both at the cellular and tissue levels. We propose that exploring mechano-metabolic interplay may reveal new targetable vulnerabilities.

## Cancer mechanics and metabolism in the brain

Mechanical signals from the brain and tumor microenvironment (TME) modulate cell and tumor mechanics and influence cell metabolism to promote the aggressiveness of cancer (42, 43). Cancer cells mechanically interact with their environment *via* mechanotransduction by converting mechanical cues into biochemical outputs (44). Mechanical stresses—sensed through conserved mechanotransduction pathways—can alter the metabolism and behavior of cancer cells and can cause cancer cells to attain stem-like properties, thus driving cancer progression and metastasis (42). Mechanotransduction also leads to cytoskeletal reorganization and changes in cell stiffness, another important player in the mechanical–metabolic feedback system. As shown in **Table 2**, several other factors influence the mechanical–metabolic feedback system in the body.

**Table 2:** Mechanical parameters and their mediators that impact cancer cell invasion/metastasis.

Parameter	Mediator
Intracellular viscosity and elasticity	Dynamic cytoskeletal proteins (actin, keratin, etc.), microtubule and microfilament polymerization and stability
Cell membrane viscosity and elasticity	Cholesterol content, saturated/unsaturated membrane lipid ratio
Stiffness of stromal cells	Varies with cell type and function, can be altered by tumor cell signaling
Stiffness of microenvironment	ECM content (collagen, hyaluronic acid, etc.), concentration, and degree of crosslinking

In most cancers, the mechanically and spatially heterogeneous TME induces metabolic alterations, facilitating cancer cells to dynamically tune energy generation in response to fluctuating energy needs (45). Proliferation rates and propensity for migration are higher in cancerous cells than in normal cells, which requires more energy and allows them to preferentially respond to environments with higher nutrient and energy production (46, 47). Mechanotransduction provides signals to control cell proliferation, differentiation, and death and requires the metabolism of nutrients for both energy generation and biosynthesis of macromolecules (48). Not only do these functions depend on cell mechanics, but they also largely depend on the mechanical properties and functions of the extracellular matrix (ECM) (44).

In tumors, ECM stiffness is largely governed by the deposition and crosslinking of collagen as well as the presence of hyaluronic acid (49–52). Many cell activities are influenced by the properties of the ECM, including metabolic reprogramming (47). The mechanical properties of individual single cells and the sensing of external forces induce metabolic changes in the cells, which in turn regulate cell and tissue mechanics (e.g., cytoskeletal changes, ECM production) (53). For example, normal cells in softer ECM (such as the brain, which lacks collagen and other stiffening molecules) have fewer bundled actin fibers than those near stiff ECM structures (54). Cells within soft ECM facilitate optimal glycolysis by mediating TRIM21 (tripartite motif containing-21, a ubiquitin ligase) and inducing subsequent degradation of phosphofructokinase (PFK), a rate-limiting glycolytic enzyme (54). In contrast, cells surrounded by stiff ECM have increased cell-surface tension, which has promoted highly bundled actin fibers that entrap TRIM21, rendering it inactive and increasing the rate of glycolysis (54). In most cancers, increasing ECM stiffness upregulates the number of glucose transport proteins in the cell membrane, increases glycolytic enzymes and glycose synthase activity, induces the expression of gluconeogenic genes, and enhances the pentose phosphate pathway, all of which increase cancer cell metabolism (47). Thus, changes in cell and/or ECM stiffness—influenced by microenvironmental factors and mechanobiological signaling—play an important role in cell growth, proliferation, migration, and malignancy (36, 55, 56).

While ECM stiffness and cell–ECM adhesion are important regulators of tumor cell invasion, ECM degradation through enzymes such as matrix metalloproteinases (MMPs) is also critical for cell migration (57). The acidic tumor microenvironment resulting from overactive cell metabolism is favorable for MMP activation (58). The resulting metabolic shift toward aerobic glycolysis in cancer cells supports the production of MMP2, for example, which can help clear a path through the ECM (58). Thus, metabolism contributes to protease-enabled cell migration.

The transcriptional regulators YAP and TAZ are largely responsible for the effects of ECM stiffness on cellular glucose metabolism (47). They integrate mechanical cues and responses to soluble signals and metabolic pathways to control several aspects of cell behavior, including proliferation and migration. In normal development, the Hippo pathway serves to regulate YAP/TAZ activity to control cell proliferation and stemness. Mechanosensory processes are integrated into the Hippo pathway, linking mechanical stress to the transcriptional response of the cell. For example, stiffened ECM causes actin polymerization within the cell, which inhibits the downstream Hippo pathway, allowing YAP/TAZ to migrate to the nucleus and function as transcription factors to promote proliferation (59). YAP/TAZ activation also regulates metabolic processes, promoting aerobic glycolysis and responding to local glucose levels (60). In breast tumors, YAP activation drives cancer growth and metastasis (61). YAP, which is found to be localized in the nucleus, is highly expressed in breast cancer tissues and increases metabolic transcriptional activity in breast cancer cells (62). Hence, aberrant YAP/TAZ activation can specifically promote cancer cell metastasis, such as breast cancer brain metastases (63, 64).

In contrast to ECM stiffness, cellular stiffness is primarily influenced by the structural composition of the cytoskeleton, i.e., increasing structural protein density (e.g., actin, keratins) can increase cell stiffness (37, 65). However, in response to the aberrant biomechanical tumor microenvironment, cancer cell cytoskeletal proteins undergo significant degradation and reduction, thus rendering these cells more mechanically compliant (37, 65–68). This is the case for most metastatic cancers (including breast cancers), where the rigidity of the cytoskeleton decreases with tumor progression, especially in highly metastatic cells (37, 65, 66).

Cell cytoskeletal composition and their dynamic alterations during motility can also contribute to metabolic alterations. The focal adhesion proteins activated during cell adhesion and detachment guide mitochondrial regulation and govern the rate of ATP production (48, 69). In the brain, for example, they can catabolize gamma-aminobutyric acid (GABA) to create nicotinamide adenine dinucleotide + hydrogen (NADH) for the support of biosynthetic processes for sustained proliferation and migration (8). Highly proliferative metastatic cancer cells with low adhesion, higher PI3K expression, and loss of PTEN tend to use alternative endogenous substrates for their metabolism and continued proliferation (8, 70). Thus, cytoskeletal protein polymerization and cell mechanics are intrinsically connected and can largely be affected by the microenvironment (53).

The phosphatidylinositol 3-kinase (PI3K) signaling pathway, which plays a role in the regulation of glucose metabolism and renders the cells dependent on high levels of glucose flux, is activated *via* integrin-mediated activation of focal adhesion kinase (FAK) (71–73). However, PI3K is dysregulated through various mechanisms, including loss or inactivation of the tumor suppressor PTEN, mutation or amplification of PI3K, and activation of tyrosine kinase growth factor receptors or oncogenes upstream of PI3K (73). PI3K is active in brain metastases, including those from breast cancer (71–73). Activation of the PI3K pathway initiates a cascade that results in the formation of new actin fibers and branching of existing fibers. This actin polymerization leads to protrusions at the cell membrane, such as lamellipodia and invadopodia. Mature invadopodia mediate cell interaction with and movement within their microenvironment and involve both cytoskeletal structures that facilitate cell movement and the delivery of matrix-degrading proteases to clear a path (74). There is also emerging evidence that cytoskeletal processes in turn regulate PI3K activity, potentially completing a positive feedback loop that results in cancer cell invasion (75).

In the brain, the mechanical properties of cancer cells and tumors in the metastatic environment are opposing (e.g., tumor versus host stiffness). Cellular stiffness is generally higher than that of the ECM, and the overall tumor generally softens with cancer progression (16). This undoubtedly affects and is affected by cell metabolism and may partially explain the comparatively low metastatic rate of cancerous cells from the supportive brain microenvironment to extracranial sites, particularly in the context of primary brain tumors (53, 76). We, therefore, hypothesize that due to increased glucose supply in the brain, metastatic cancer cells often migrate to the brain, while brain cancer cells preferentially invade locally rather than extravasate and metastasize to other parts of the body.

## Cancer metabolism and metastasis in the brain

**Figure 2:**
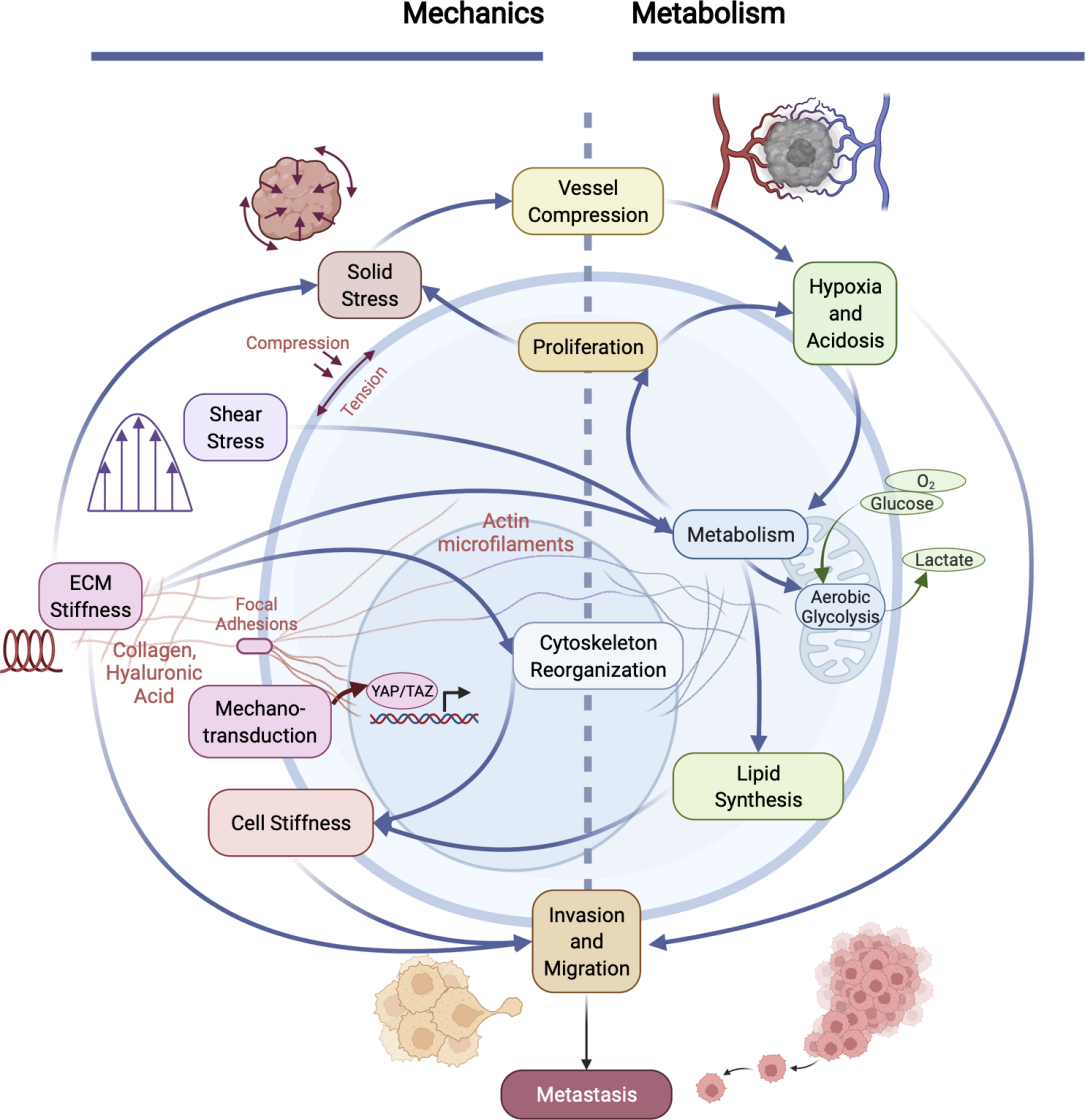
Cancer invasiveness and metastatic potential are regulated by the cooperation between aberrant cellular/tissue mechanics and altered metabolism in tumors. The physical and metabolic characteristics of tumors and their microenvironment interact in many distinct ways. Heightened solid stress (compressive and tensile) compresses tumor blood vessels, exacerbating hypoxia and acidosis within the microenvironment, which influences metabolism. Increased cell metabolism (e.g., aerobic glycolysis and lactate production) enables cell proliferation, which in turn causes an increase in solid stress. Metabolism is also influenced by increased fluid shear stress and elevated ECM stiffness, which results in altered cytoskeletal organization and reduced cancer cell membrane stiffness. Mechanical properties and forces are sensed *via* mechanotransduction pathways (e.g., focal adhesion kinase (FAK), resulting in translocation of YAP/TAZ to the nucleus which influences many cells physiological properties). Cell stiffness is also influenced by lipid metabolism in cancer cells. Together, co-regulated metabolic and mechanical alterations in cancer cells directly promote invasion and metastasis.

Cancer cells consume excess nutrients and energy compared with benign cells. The Warburg effect alters cancer cell metabolism by increasing glucose uptake and the fermentation of glucose to lactate (77, 78) (**Figure 2**). This process, known as aerobic glycolysis, is less efficient than the complete mitochondrial respiration cycle that occurs in normal cells, but it may provide comparable amounts of energy and even confer a survival advantage to cancer cells. In glioblastoma, for example, which is the most common and malignant primary adult brain tumor, a metabolic shift is observed toward aerobic glycolysis (13). Glioma cells adapt to maximize their ability to synthesize substrates for membrane lipids, nucleic acids, and proteins for increased proliferation and migration (13). In breast cancer brain metastases, fatty acid synthesis is elevated compared to the metabolism at extracranial sites, resulting in a site-specific metabolic dependency (79). A recent study by Parida et al. showed that brain-tropic Her2+ breast cancer cell metabolic diversity and plasticity shape their metastatic fitness (80). These cells outcompete proximate cells in the brain for glucose uptake, metabolize lactate, hinder immune surveillance, and successfully seed brain metastases (80). This metastatic process requires *de novo* serine synthesis to proliferate in the brain microenvironment due to reduced amino acid levels in the brain relative to the plasma (81). However, because biochemical factors such as oxygen and glucose influence cell migration and metabolism, a well-nourished brain promotes cancer cell seeding and invasion (82). Initial metabolic plasticity also supports metastasis to and survival in the brain, where cancer cells lose some of their metabolic flexibility compared to the primary tumor upon colonization (82).

Intrinsic and extrinsic factors affect cancer metabolism and metastasis (8, 83). Intrinsically, mutations and changes in gene expression support metabolic shifts and can directly alter the levels or activity of metabolic enzymes within cancer cells (8). The loss of tumor suppressor genes such as phosphate and TENsin homolog deleted on chromosome 10 (PTEN) correlates with a significant increase in the risk of brain metastases in melanoma, breast, and lung cancer patients (83–86). Extrinsically, interactions with the extracellular matrix, surrounding cells, and available nutrients affect cell metabolism. For example, ECM alterations due to primary therapies (radiotherapies and chemotherapies) increase metabolism and energy production (ATP and GTP) and can create migratory tracts to promote intracranial tumor migration, invasion, and recurrence (87–89). Since about 20% of glucose-derived energy products in the body are consumed in the brain, the local rates of glucose supply and demand provide an ideal nutrient-rich environment to fuel the growth of primary and metastatic tumors (90). It has been recently shown that breast cancer metastases feature higher expression of glycolysis-related proteins (Glut-1, hexokinase II, CAIX, and MCT4) in the brain than in other organs (bone, liver, or lung) (91). Due to enhanced gluconeogenesis and glutamine oxidation (77), brain metastatic breast cancer cells have also been shown to develop the ability to survive and metastasize independently of glucose availability.

Breast cancer cells that metastasize to the brain have a genetic predisposition for adaptability and the ability to crosstalk with host cells, influencing *de novo* metabolic changes (92). For example, during early metastatic brain colonization, the blood–brain-barrier (BBB) is selectively disrupted *via* cancer cell trafficking and the inhibition of the docosahexaenoic acid (DHA) transporter expressed by endothelial cells. This loss can induce BBB leakage, reduce DHA transport, and alter metastatic lipid metabolism (83, 93). Because glutamine and glutamate are stored in the brain microenvironment, cancer cells also use these amino acids to support their continuous proliferation and biosynthesis of macromolecules (94). The PTEN pathway is suppressed in breast metastatic cells in the brain microenvironment by astrocytes *via* the activity of exosome-delivered miRNAs that inhibit PTEN expression, thus promoting tumor growth and progression (83). Hence, the metabolic soil of the brain is primed to support the growth of the metastatic cancer cell seeds (95).

Cancer cells also increase the synthesis of cholesterol, an integral part of the cell membrane (96). This is a common characteristic of breast cancer cells, as cholesterol must be constructed into new cell membranes in dividing cells (97). However, cholesterol synthesis is particularly upregulated in the brain, in part because cholesterol cannot cross the BBB (96). It has been shown that both tumor tissues and individual tumor cells have increased membrane cholesterol levels, across a range of cancer types (98). While there are contradictory findings on the effects of membrane cholesterol and cholesterol depletion in cancer cells, membrane cholesterol plays an important role in cell membrane stiffness (98). Brain metastatic cancer cells also increase total fatty acid content to support membrane biosynthesis, though unsaturated fatty acid synthesis is decreased (96, 98). Interestingly, unsaturated fatty acids lead to a more rigid cell membrane, i.e., a decrease in unsaturated fatty acid content may correspond to a decrease in cancer cell fluidity (99). Together, cholesterol and lipid synthesis provide yet another link between the biological and mechanical characteristics of brain metastatic cells. Indeed, the brain appears to provide a metabolic microenvironment that mechanically reinforces the cellular structure of metastatic cancer cells.

## Cancer mechanics and metastasis in the brain

Numerous studies have shown the interdependency of mechanics and metastatic behavior of cancer cells (65, 66, 100, 101). The mechanical properties (e.g., stiffness, viscosity) of most cancer cells, including those in the breast, are lower than their counterparts in normal cells (67, 102, 103). Most cancerous cells have altered viscoelastic properties (lower stiffness and viscosity) that allow cells to move easily through the interstitium and tumor microarchitecture on their way to metastatic sites (104). In contrast, brain cancer cells within softer tumors may be less likely to systemically metastasize and may be partially constricted by the stiffer tissue of the surrounding host brain compared to the tumor stiffness, as well as the tumor mechanical stresses housed and amplified within the skull, all of which reduce the ease of migration (105). As described earlier, at the cellular level, brain cancer cells are stiffer than normal glial cells (76).

Besides well-known mechanical abnormalities such as cell and matrix stiffness and interstitial fluid pressure, tumors also generate solid stress due to the solid elements of the tumor (30). These solid stresses promote tumor progression and hinder the delivery and efficacy of anti-cancer therapies by compressing the blood vessels and contributing to intratumoral hypoxia (30). Growth-induced stresses enhance epithelial-to-mesenchymal (EMT) transition and cancer cell migration, in part *via* activation of β-catenin, AKT, and Erk pathways, all of which are metabolically related (105, 106). Notably, reducing solid stress (e.g., *via* angiotensin receptor blockade) in breast cancer mouse models reduces lung metastatic burden (107). However, direct links between this mechanical force and cancer cell membrane stiffness and metabolic activity have yet to be determined. Nevertheless, altered mechanics at the cellular and tissue levels can drive cancer metastasis.

## Discussion

The interplay of mechanics and metabolism is largely unexplored but undoubtedly plays a major role even in the early stages of the metastatic cascade from primary sites such as breast tumors. Hypoxia and acidosis in the primary tumor microenvironment emerge due to rapid cell division, a poorly functioning vasculature, and high rates of glycolysis and lactate production in tumor cells (108). These conditions promote metabolic pathway alterations, including a switch from oxidative phosphorylation to aerobic glycolysis, to aid cancer cell proliferation (109, 110). The need for higher energy levels causes these cells to invade nearby and distal organs (e.g., the brain) where glucose levels are high and can sustain cancer growth and spread. These aggressive primary breast cancer cells have low membrane stiffness and viscosity compared to normal epithelial cells, so they can easily detach from their substrate and intravasate (65, 66, 68). The reduction in cancer cell membrane stiffness is in part due to the interactions between cell metabolism and cytoskeletal structure (109). Resistance of the cytoskeleton in response to altered mechanical cues in variable microenvironments can enable the altered energy metabolism, e.g., the persistence of high glycolytic rates in cancer cells despite chronic mechanical changes in the tumor tissue (109, 110).

The invasion occurs from the primary invasive tumor site (breast), through the circulation (blood vessels), to the secondary metastatic site (brain). Cancer cells experience distinct mechanical and metabolic microenvironments at each stage, leading to biophysical adaptations. While in circulation within the blood vessel, metastatic cancer cells experience varying mechanical forces such as shear stress, which can activate genetic programs associated with cytoskeletal remodeling and altered cell–cell adhesion (36). Shear stress also activates ATOH8, a fluid mechanosensor that transcriptionally promotes glycolysis and reduces reactive oxygen species (ROS) (111). This promotes cancer cell survival in the bloodstream by enabling metabolic flexibility. Notably, most cancer cells do not survive the circulation, which may be due in part to the varying mechanical microenvironment and/or altered metabolism within the blood vessel (112, 113). Krog et al. also suggested that the mechanical fragility of circulating cancer cells is not necessarily due to the magnitude and duration of exposure to fluid shear stresses during circulation but possibly due to other secondary causes such as their exposure to immune attack in the blood vessels and lack of matrix attachment (113). They also concluded that these cancer cells may be as likely to withstand hemodynamic stresses as other blood cells during circulation (113). Cancer cells that survive the circulation, however, acquire energy *via* alternate carbon sources than they do in the solid tumor microenvironment (114).

Once in the brain, breast cancer cells may assume brain-like properties to survive, e.g., by using GABA to synthesize NADH for energy production (115). Exposure to new mechanical properties and forces drives further metabolic and mechanical changes during metastasis, including the stiffening of the disseminated cancer cell membrane relative to those in the primary breast tumor (116, 117). Metabolically (e.g., *via* production of cytoskeletal proteins), mechanical cues from the tumor microenvironment can lead to increased cell stiffness (110). Reinforcement of the cytoskeletal structures and rigidity of the cell viscoelasticity in the brain may prevent secondary brain tumors from seeding tertiary extracranial metastases (or primary brain tumors from seeding initial secondary metastases). Further exploration into the co-regulation of mechanics and metabolism in the cerebral microenvironment may provide new insights and reveal new targetable vulnerabilities for breast cancer brain metastases.

## Conclusion

The interaction between mechanics and metabolism is multifaceted and plays a pivotal role in cancer progression, as seen in the case of breast cancer metastasis to the brain. The brain microenvironment provides a favorable mechanical and metabolic microenvironment for disseminated cancer cells, helping promote tumor growth and invasion. However, it is challenging to fully understand the relationship between mechanics and metabolism due to heterogeneity within a tumor, between different tumors in one patient, between different patients of the same tumor type, and between different patients with different cancers. Current methods for measuring cell and tissue stiffness, such as atomic force microscopy and magnetic resonance elastography, could be improved to provide more accurate and high-resolution information. Understanding the complete metabolic milieu of a cell requires the acquisition of a complex range of metabolomics data, which is time-sensitive and cost-intensive and may not always capture the true *in vivo* (particularly dynamic) state. Further advancements in metabolic analysis methods combined with mechanical probing would aid in revealing the dynamic and heterogeneous metabolic states within a tumor and how they correlate with metastasis and patient outcomes. In the future, information about the mechanics and metabolism of patient-derived cancer cells could also serve as valuable biomarkers of metastasis.

## Data availability statement

The original contributions presented in the study are included in the article/supplementary material. Further inquiries can be directed to the corresponding author.

## Author contributions

KO and MD conceptualized and wrote the manuscript. KO and AB revised the manuscript and created the figures. All authors listed have made a substantial, direct, and intellectual contribution to the work and approved it for publication.

## Acknowledgments

The authors thank Guest Editor Dr. Gino B. Ferraro for the invitation to contribute this article to the special issue. Figures were created with BioRender.com. This work was supported by the National Cancer Institute (NIH/NCI K22 CA258410 to MD).

## Conflict of interest

The authors declare that the research was conducted in the absence of any commercial or financial relationships that could pose potential conflicts of interest.

## Publisher’s note

All claims expressed in this article are solely those of the authors and do not necessarily represent those of their affiliated organizations, or those of the publisher, the editors and the reviewers. Any product that may be evaluated in this article, or claim that may be made by its manufacturer, is not guaranteed or endorsed by the publisher.
